# An Examination of Psychopathology and Daily Impairment in Adolescents with Social Anxiety Disorder

**DOI:** 10.1371/journal.pone.0093668

**Published:** 2014-04-01

**Authors:** Franklin Mesa, Deborah C. Beidel, Brian E. Bunnell

**Affiliations:** University of Central Florida, Orlando, Florida, United States of America; The University of Texas at Austin, United States of America

## Abstract

Although social anxiety disorder (SAD) is most often diagnosed during adolescence, few investigations have examined the clinical presentation and daily functional impairment of this disorder exclusively in adolescents. Prior studies have demonstrated that some clinical features of SAD in adolescents are unique relative to younger children with the condition. Furthermore, quality of sleep, a robust predictor of anxiety problems and daily stress, has not been examined in socially anxious adolescents. In this investigation, social behavior and sleep were closely examined in adolescents with SAD (*n* = 16) and normal control adolescents (NC; *n* = 14). Participants completed a self-report measure and an actigraphy assessment of sleep. Social functioning was assessed via a brief speech and a social interaction task, during which heart rate and skin conductance were measured. Additionally, participants completed a daily social activity journal for 1 week. No differences were observed in objective or subjective quality of sleep. Adolescents with SAD reported greater distress during the analogue social tasks relative to NC adolescents. During the speech task, adolescents with SAD exhibited a trend toward greater speech latency and spoke significantly less than NC adolescents. Additionally, SAD participants manifested greater skin conductance during the speech task. During the social interaction, adolescents with SAD required significantly more confederate prompts to stimulate interaction. Finally, adolescents with SAD reported more frequent anxiety-provoking situations in their daily lives, including answering questions in class, assertive communication, and interacting with a group. The findings suggest that, although adolescents with SAD may not exhibit daily impaired sleep, the group does experience specific behavioral and physiological difficulties in social contexts regularly. Social skills training may be a critical component in therapeutic approaches for this group.

## Introduction

Social fears are relatively common, as 24.1% of respondents in a recent U.S. survey reported at least one lifetime social fear [1]. Social anxiety disorder (SAD) is typified by a pattern of excessive fear of social situations or performances in which an individual may be scrutinized by others [2]. Public speaking, meeting new people, and speaking up during a meeting/class are common distressful situations for people with SAD [1], [3]. Although additional psychiatric disorders often co-occur among people with SAD [1], [4], [5], for many individuals the onset of SAD predates the onset of comorbid psychiatric conditions [6].

The prevalence of SAD within the general population is among the highest of all the anxiety disorders. Recent estimates have placed its 12-month and lifetime prevalence at approximately 7% and 12% in community samples, respectively [7], and without treatment, the course of the disorder spans decades [8]. Although initial research focused on adult samples, the average age of onset of SAD is during adolescence [9], typically between 15 and 16 years of age [3], [10]. The disorder also occurs in children as young as age 8 [11]. Within youth specifically, observed prevalence of SAD ranges from approximately 1% [12] to 5.4% [13] in community samples and even higher rates have been reported from clinical samples [14].

Preliminary descriptions of the psychopathology and clinical presentation of SAD largely excluded children and adolescents [11].When interests shifted to SAD in youth, studies initially focused on pre-adolescent children, demonstrating consistency in clinical presentation between children and adolescents [11], [15], [16], [17].

Ironically, relatively few studies have examined the psychopathology of SAD exclusively in adolescent samples, even though adolescence marks the average age of onset. The majority of studies of SAD psychopathology in youth utilize samples combining children and adolescents. However, the small sample of extant comparisons of adolescent SAD and childhood SAD reveal features unique to each group. Adolescents with SAD reported significantly higher fear ratings across all situations and reported significantly higher ratings of avoidance than children with the same disorder [18]. A significantly larger percentage of adolescents endorsed at least moderate avoidance of social situations, including “asking a teacher a question,” “attending social activities,” “working with a group,” “walking in the hallways,” and “dating.” Furthermore, adolescents endorsed more loneliness than young children, suggesting a greater impact of their fear on their social functioning when compared to younger children. In contrast, children had longer speech latencies during a role-play task and were rated as significantly less skilled as well as more anxious than adolescents during social interaction and speech. Therefore, even if adolescents have more severe clinical symptoms and may be suffering more overall impairment, it appears that they are able to socially engage somewhat more effectively than their younger counterparts.

As expected, epidemiological and clinical samples of adolescents with SAD report excessive fear and avoidance in social and performance situations [19], [20]. When encountering these situations, adolescents with SAD frequently report increased physiological arousal [20], [21]. Perceived physiological arousal, however, often does not coincide with actual physiological reactivity. In one investigation, despite differences in perceived physiological reactivity, there were no group differences between adolescents with SAD or no disorder on measures of heart rate reactivity or blood pressure change when interacting with another person or giving a speech [21]. Similar findings were reported when heart rate reactivity was assessed among normal controls, subclinical socially anxious adolescents, and adolescents with SAD [22]. These findings suggest that perceptions of increased physiological arousal produce significant distress among adolescents with SAD, though they may not experience actual increased physiological arousal in laboratory settings. However, assessing more than one channel of physiology may be necessary to accurately determine the existence of increased physiological response, as these measures are not pure measures of sympathetic activation. When the HR signal was examined more closely in children with high and low social anxiety, those with high social anxiety manifested higher sympathetic activation during baseline [23].

Consistent with other developmental stages, SAD in adolescents is associated with social skill deficits such as reduced facial gaze and increased response latencies during social interactions [24]. Furthermore, social anxious adolescents are rated by independent raters and rated themselves less effective (i.e., less skilled) than normal controls during read-aloud tasks, an impromptu speech, and role-play tasks [19], [25]. However, there are potential drawbacks of previous social skill examinations. For example, one assessment of social skill [19] uses a structured role-play task that may not represent typical adolescent interactions, potentially reducing external validity. Further, assessment of social skill consisted mostly of broad ratings (i.e., effectiveness, social skillfulness, and assertiveness) that may be difficult to compare across studies [19], [25]. An assessment of more specific behaviors, such as duration of speaking and frequency of questions and comments during a social interaction, would significantly enhance our knowledge of social behaviors in youth with SAD.

Collectively, these data highlight our limited understanding of the clinical presentation of social anxiety disorder in adolescents. Even less is known about how the hallmark features of the disorder (e.g., poor social performance and physiological reactivity in social situations) affect daily stress level and functioning in social interactions, leisure time, and school/work [19], [20].

Quality of sleep is an additional important correlate of daily stress level in adolescents with anxiety disorders, given the bidirectional relationship between sleep problems and anxiety difficulties [26]. Interest in the quality of sleep of youth with anxiety disorders has increased in recent empirical work. While diary and self-report assessments of stress can be biased or minimized, sleep is a more objective metric of daily stress; however, the literature on sleep and SAD is mixed. Several studies have demonstrated a relationship between clinical anxiety and sleep difficulties among adult samples, with mixed findings on the relationship between SAD and quality of sleep [27], [28], [29], [30]. Among youth samples, sleep problems have been more strongly associated with generalized anxiety disorder or separation anxiety disorder than SAD [31], [32]. For instance, youth diagnosed with generalized anxiety disorder in one study reported the greatest rate (87%) of difficulty sleeping, while 27% of youth diagnosed with SAD reported difficulty sleeping [33]. In a study using an objective assessment of quality of sleep [34], youth diagnosed with anxiety disorders had poorer quality of sleep than youth diagnosed with major depressive disorder as assessed by polysomnography. Specifically, youth with anxiety disorders experienced significantly more awakenings and spent significantly longer periods of time awake during the night. Nevertheless, a small proportion of participants in this investigation had a primary diagnosis of SAD and statistical analyses were not reported for individual diagnostic groups. Thus, it is unclear from the extant literature whether adolescents with SAD experience both objective and subjective sleeping difficulties, particularly in anticipation of or following an anxiety producing social event (e.g., an upcoming presentation or an embarrassing situation during the day) [32].

Given the limitations in the current literature, this study aimed to further elucidate the clinical presentation as well as the functional impairment of SAD in adolescents, data that may be used subsequently to evaluate the effectiveness of psychosocial interventions. In this investigation, adolescents with SAD or no disorder were compared in overt social behavior and physiological reactivity during a social interaction task and a performance task (heart rate [HR] and electrodermal skin activity [EDA]). Additionally, objective and subjective quality of sleep were examined.

The study had the following hypotheses:

- Adolescents with SAD will exhibit poorer overt social behaviors during each of two social tasks than controls.
- Adolescents with SAD will exhibit heightened physiological response than controls during each of two social tasks.
- Adolescents with SAD will report poorer daily social functioning.
- Adolescents with SAD will exhibit poorer objective and subjective quality of sleep than controls.

## Method

### Participants

Participants were recruited via community advertisements placed through the Central Florida targeting “friendly” or “shy” adolescents seeking treatment aged 13 to 17 years old. To be included in the study, socially anxious adolescents had to have a primary diagnosis of SAD. Adolescents with suicidal ideation or a primary diagnosis other than SAD were excluded and referred to appropriate service providers in the community. Adolescents with secondary or comorbid diagnoses were included as long as SAD was the primary diagnosis. Sixteen socially anxious adolescents aged 13 to 17 (*M* = 14.81, *SD* = 1.47) were included in the SAD group. Secondary diagnoses in the socially anxious group consisted of generalized anxiety disorder (18.75%), dysthymic disorder (12.5%), major depressive disorder (12.5%), specific phobia (6.25%), and selective mutism (6.25%). Fourteen control (friendly) adolescents ranging in age from 13 to 17 (*M* = 15.07, *SD* = 1.54) were included who did not meet diagnostic criteria for any current Axis I diagnosis. The groups did not differ significantly in age, *t*(28) = .470, *p* = .642 or household income, *t*(28) = −.265, *p* = .793. Although the control group was more balanced across ethnicities, both groups consisted predominantly of participants self-identified as White (SAD: 68.8%, controls: 42.9%). The SAD group also included Latinos/Hispanics (25%) and 1 Asian American (6.2%). Latinos/Hispanics made up 21.4% of the control group and African Americans made up 35.7%. The groups did not differ statistically in ethnic composition, *χ* ^2^(3) = 7.514, *p* = .057. However, there were more females in the SAD group than the control group and more males in the control group than the SAD group, and the difference was statistically significant, *χ* ^2^(1) = 5.129, *p* = .024.

### Diagnostic Assessment

Participants and their parents were assessed separately by doctoral-level clinical psychology students with the Anxiety Disorders Interview Schedule – Child/Parent Version [35], a semistructured diagnostic interview. Interviewers determined diagnoses by considering information provided by both the parents and the adolescents. Diagnoses were given if either interviewee endorsed a disorder per ADIS-C/P guidelines. There were no diagnostic disagreements between adolescents and their parents. Twenty percent of the parent and child interviews were assigned to a second blinded rater to determine inter-rater reliability for the primary diagnosis, which was excellent (κ = 1.00).

### Overall Assessment Strategy

Each adolescent completed a comprehensive assessment designed to assess the functional impairment of SAD in an adolescent sample. This protocol was approved by the University of Central Florida Institutional Review Board. At least one parent, in addition to the participating adolescent, reviewed the University of Central Florida Institutional Review Board-approved consent form and was afforded the opportunity to address any concerns with the first author. Written consent to participate was obtained from the adolescent and his or her parent. Self- report and behavioral assessments of social and global impairment as described below.

#### Behavioral assessment

Two counterbalanced behavioral tasks were used to assess social functioning – one to assess behavior during a social interaction and a second to assess behavior during a public performance. The behavioral tasks were digitally recorded using the Noldus Observer System. Specific behaviors were coded for each task by undergraduate raters who were trained to acceptable reliability by the first author. Twenty-five percent of the recorded behavioral tasks were coded by a second rater for reliability.

The social interaction task (SIT) examined social skill in a situation that adolescents may encounter in daily living. Study participants joined an undergraduate confederate playing with an entertaining video game console (Nintendo Wii) for 10 minutes. The undergraduate confederates were instructed to greet the participant and provided with 5 specific prompts (e.g., an invitation to play Wii and 4 additional questions) to use throughout the task to ensure that each interaction was standardized. The additional questions were only given if the participant had not spoken in the previous two minutes, excluding responses to the previous prompt. Participant behaviors coded during this task were: speaking latency, time to first spontaneous verbalization (e.g., statement or question not in response to a question), number of spontaneous comments, number of questions asked, and duration of speech. The intraclass correlations for all SIT coded behaviors were excellent (all ICC ≥.98).

The speech performance task (SPT) assessed performance, anxiety and avoidance behaviors while speaking in front of a small audience. Prior to beginning the SPT, participants were given 7 possible topics (e.g. favorite subjects in school, favorite movies) to discuss and allotted 3 minutes to prepare. Participants were instructed to speak for up to 10 minutes and informed that the task would last at least 3 minutes. Participants were notified via a blue light that 3 minutes had elapsed. Speaking after this point was optional, and participants were given a “STOP” card to display if they wanted to end the task. Following the 3-minute preparation period, participants were removed from the study room briefly while four audience members entered, most frequently comprised of 2 female and 2 male undergraduate research assistants who did not interact with participants during the task. Participants returned to the study room after the audience was in place and delivered the speech, during which they were allowed to refer to the topic sheet. The task was ended early if participants exhibited severely overwhelming distress. During the SPT, the following behaviors were coded: speaking latency (seconds), duration of speaking (seconds), escape (task ended prior to 3 minutes), and number of topics discussed. The intraclass correlations for all SPT coded behaviors were excellent (all ICC ≥.94).

#### Physiological assessment

In addition to social behavior, HR was assessed during both behavioral tasks and EDA was assessed during the SPT only as participation in the video game created too many artifacts for accurate scoring of EDA during that task. The MindWare Ambulatory system was used to collects physiological data wirelessly. Three electrodes were placed on the torso to measure HR and two were placed on the fleshy areas of participants' non-dominant hand to assess EDA. The electrodes were connected to a PDA that wirelessly transmitted data to acquisition software on a desktop computer. A 10-minute baseline period preceded the first behavioral task, and a 5-minute recovery phase was implemented prior to the second behavioral task to allow physiological activity from the first task to return to pre-task levels. All physiological data were examined and movement artifact was removed.

#### Self-report of distress

Subjective distress was assessed by self-ratings of anxiety recorded prior to and following each behavioral task using a 9-point Likert scale ranging from 0 (i.e., completely calm and relaxed) to 8 (i.e., extreme fright—terror).

#### Daily social activity – anxiety journal

Participants were given a list of social situations derived from the SAD section of the ADIS-C/P and asked to log their participation/avoidance of daily social encounters. Participants rated their perceived distress on a 9-point scale ranging from not anxious at all (0) to extremely anxious (8). Socially anxious events were defined as social interactions resulting in anxiety rating of at least 3.

#### Quality of sleep

Sleep actigraphy, using Micro Sleep Watch actigraphs (Ambulatory Monitoring, Inc., New York), estimated quality of sleep. These actigraphs resemble wrist watches and include an event-marker function that allows users to identify distinct periods (i.e., periods of sleep). Participants wore an actigraph on the non-dominant wrist for a period of at least 7 days and nights while maintaining their daily schedule. Participants were instructed to use the event marker function when attempting to fall asleep and again when waking in the morning. Participants were also instructed to maintain a sleep log to note bedtimes, waking times, and events that may have affected sleep quality (e.g., illness or significant life stressors). Raw data collected by the actigraphs were organized into 1-minute epochs and were analyzed by accompanying ActionW software. The two variables from actigraphy selected for analyses were wake minutes (duration of time awake during the designated period of time) and sleep efficiency (percentage of time scored as sleep [as opposed to awake] between onset of sleep to offset of sleep). These indices of sleep have demonstrated the highest reliability in adolescent samples [36]. Additionally, participants completed the School Sleep Habits Survey (SSHS) [37], [38], a self-report measure of quality of sleep developed specifically for use with adolescent samples. The SSHS assesses other aspects of functioning as well (e.g., depressive symptomology and academic functioning) and contains 63 items with varied response options (e.g., yes and no responses as well as numerical responses). Items on the SHSS address multiple aspects of sleep, including bed- and wake times and sleep duration. Two subscales of SHSS were the primary focus: the Sleepiness Scale (SS) that consists of 10 items on the SHSS examining difficulties staying awake or falling asleep in different situations, and the Sleep/Wake Problems Behavior Scale (SWPB) that contains 15 items measuring recent erratic sleep/wake times. These subscales have demonstrated acceptable internal reliability (.70 and.75, respectively) [37] among adolescent samples. Furthermore, the SHSS has been shown to be a valid measure of sleep habits in adolescents as evidenced by correlations with actigraphy data and self-report sleep on total sleep time (.53 and .61, respectively), sleep onset time (.70 and .76, respectively), and sleep offset time (.77 and .71, respectively) [38]. The internal consistency for the SS was acceptable (Cronbach's alpha  = .65) in the present sample; however, the SWPB demonstrated poor internal consistency (Cronbach's alpha  = .54) and was withheld from the statistical analyses.

## Results

Initial analyses of demographic variables revealed a significant difference for sex ratio; however, the outcomes were largely consistent with or without sex as a covariate. Therefore, sex was not used as a covariate to avoid discarding meaningful variance. Group differences were examined with multivariate analysis of variance on selected groups of dependent variables, followed by ANOVA when the overall MANOVA statistic was significant at p<.05.The correlations among variables targeted in each MANOVA were examined. In two of the three instances of strong correlations, one of two highly correlated variables was dropped to prevent multicollinearity (wake minutes from actigraphy and avoided social activities from the daily journal). In the third instance among SIT coded variables, a step-down analysis was implemented as described by Tabachnick and Fidell [39]. Briefly, the step-down analysis involves prioritizing the dependent variables, then performing an initial ANOVA with the primary dependent variable. The remaining dependent variables are examined in a series of ANCOVAs where the higher-priority dependent variables are entered as covariates. Social behaviors and physiological variables were assessed separately for each behavioral task. Given that many individuals experience some discomfort during a public speech task, analyzing data from the tasks in one MANOVA could have masked important differences in the social interaction.

### Quality of Sleep

The SS score from the SSHS as well as the actigraph-measured variable were examined in a 2 (group) x 2 (quality of sleep measures) MANOVA. One participant in the NC group did not complete the actigraphy assessment. Overall quality of sleep did not differ between SAD and NC participants, F(2, 26) = 2.104, p = .142, ηp^2^ = .139. Means and standard deviations for each of the sleep variables are presented in Table 1.

**Table 1:** Descriptive and ANOVA statistics for sleep and behavioral variables.

Variable	Sub-variable	NC (N = 14)	SAD (N = 16)	*F*	*d*	ηp2
		M (SD)	M (SD)			
Sleep Efficiency		91.46 (3.90)	94.29 (3.50)			
SS Total		13.92 (3.62)	14.12 (2.58)			
Completed activities		60.36 (25.08)	43.53 (18.35)	4.294	.048	.137
Anxious events		0.38 (0.47)	1.06 (0.41)	17.85	<.001	.398
SIT distress ratings	Pre-task	1.43 (1.40)	2.81 (1.97)	4.774	.037	.128
	Post-task	.93 (1.14)	2.91 (2.22)	4.124	.052	.132
Speaking duration		46.11 (33.97)	40.79 (48.57)	.115	.737	.004
Prompts		1.14 (1.41)	2.33 (1.63)	7.588	.011	.226
SIT-speaking latency		14.28 (32.88)	40.60 (95.19)	.247	.624	.010
Questions		7.07 (6.68)	2.20 (2.11)	3.228	.085	.119
Spontaneous comments		8.14 (8.02)	7.53 (12.04)	.011	.917	.000
SVL		97.80 (157.72)	271.51 (268.84)	1.132	.299	.049
SPT distress ratings	Pre-task	1.21 (1.42)	2.28 (1.71)	3.384	.076	.108
	Post-task	4.21 (2.01)	6.62 (1.54)	10.278	.003	.276
SPT-speaking latency		1.75 (2.02)	4.42 (4.76)	3.733	.064	.126
Duration of speech		167.75 (38.38)	83.09 (44.83)	28.806	<.001	.526
Topics discussed		3.93 (2.92)	4.5 (2.41)	.319	.577	.012

Sleep efficiency values are in percentage. Latency and duration values are in seconds. SS: School Sleep Habits Survey Sleepiness Scale; SIT: Social Interaction Task; SVL: Spontaneous verbalization latency; SPT: Speech Performance Task.

### Daily Social Activity

Daily social functioning assessed via the anxiety journal was examined in a 2 (group) x 2 (frequency of journal completed activities and socially anxious events) MANOVA. One SAD participant did not complete the anxiety journal. A log transformation was performed on the socially anxious events variable, as the distribution of frequencies was not initially normally distributed. The overall MANOVA revealed a main effect for group indicating that SAD and NC participants differed in their report of daily social activity and distress, *F*(2, 26) = 12.849, *p* = <.001, η_p_^2^ = .497. Follow-up analyses indicated that participants with SAD reported completing fewer social activities relative to NC participants, *F*(1, 27) = 4.294, *p* = .048, η_p_^2^ = .137. Further, the SAD group reported a greater frequency of social activities that caused at least mild to moderate anxiety, including speaking to a stranger, interacting with a group, and answering questions in class, *F*(1, 27) = 17.847, *p* = .<001, η_p_^2^ = .398. Descriptive statistics for the journal data are found in Table 1.

### Behavioral Assessment

#### Self-report of distress in social interaction and speech task

Self-report of distress during the behavioral tasks was examined using two 2 (group) x 2 (anxiety ratings pre- and post-behavioral task) repeated measures ANOVAs ratings. For the SIT, there was an overall main effect of group for self-ratings of distress, *F*(2, 27) = 4.715, *p* = .018, η_p_^2^ = .259. Subsequent analyses indicated that there were significantly higher pre-SIT distress ratings in participants with SAD, *F*(1, 28) = 4.774, *p* = .037, η_p_^2^ = .146. Participants with SAD trended toward greater distress ratings following the SIT as well, *F*(1, 27) = 4.124, *p* = .052, η_p_^2^ = .132. There was also an overall effect of group for SPT-related distress ratings, *F*(2, 27) = 7.392, *p* = .003, η_p_^2^ = .354. While pre-SPT distress ratings did not differ between groups, participants with SAD reported significantly greater distress following the SPT than NC participants, *F*(1, 27) = 10.278, *p* = .003, η_p_^2^ = .276. Means and standard deviations for anxiety ratings pre- and post-behavioral tasks are contained in Table 1.

#### Social behaviors

One SAD participant did not speak during the SIT, and two participants with SAD did not speak during the SPT. These participants were withheld from the analyses of the social behaviors during the tasks. A 2 (group) x 3 (three coded behaviors during the SPT) MANOVA assessed group differences during the SPT. A chi square was used to examine differences in escape behavior (yes/no) between groups only during the SPT, as all participants completed the entire 10-minute SIT. Data from both analogue social tasks are contained in Table1.

The SIT social behaviors were prioritized as follows: speaking duration, prompts, speaking latency, questions, spontaneous comments and spontaneous verbalization latency. Participants with SAD received more confederate prompts relative to NC participants, *F*(1, 26) = 7.867, *p* = .011, η_p_^2^ = .226. The groups did not differ on measures of speaking duration, speaking latency, questions asked, spontaneous comments or spontaneous verbalization latency. Chi square analyses indicated that participants with SAD were significantly less likely to make any spontaneous verbalizations during the 10-minute task (6 SAD versus 1 NC), *χ* ^2^(1) = 3.846, *p* = .050.

For the SPT, there was a significant overall effect for group on the coded social behaviors, *F*(3, 24) = 10.413, *p* = <.001, η_p_^2^ = .566. Follow-up analyses indicated that participants with SAD trended toward longer speaking latencies, *F*(1, 26) = 3.733, *p* = .064, ηp^2^ = .126, and spoke for shorter durations than NC participants, *F*(1, 26) = 28.806, *p*<.001, ηp^2^ = .526; however, number of speech topics discussed did not differ between groups. Finally, an examination of escape behavior during the SPT revealed that participants with SAD were significantly more likely to discontinue the task prior to the 3-minute mark (8 SAD versus 0 NC), *χ* ^2^(1) = 9.545, *p* = .002.

#### Physiological response

Group differences in task HR were examined with 2 (group) x task minutes repeated measures ANCOVAs, covarying for baseline HR (final minute of baseline). Mean skin conductance (MSC) was selected to represent EDA, and similar analyses were conducted to assess differences in MSC during the SPT only. The quantity of EDA skin conductance responses (SCR), which are rapid increases in EDA of at least .05 microsiemens, per minute were evaluated with an independent-samples t-test.

Two socially anxious participants did not have HR data available for either behavioral task due to equipment malfunction. One socially anxious participant also did not have EDA data available for the SPT due to equipment malfunction. Four additional participants with SAD discontinued the SPT too quickly to be included in the physiological analyses. SPT physiological data for the remaining participants were reduced to 30-second means and analyzed through 3 minutes, as available data decreased dramatically after this point due to participants' decision to end the task. Two participants with SAD did not exhibit any speaking behavior during the speech task. Identical analyses were performed while excluding the two participants that did not speak, and the physiological outcomes were unaffected.

**Figure 1:**
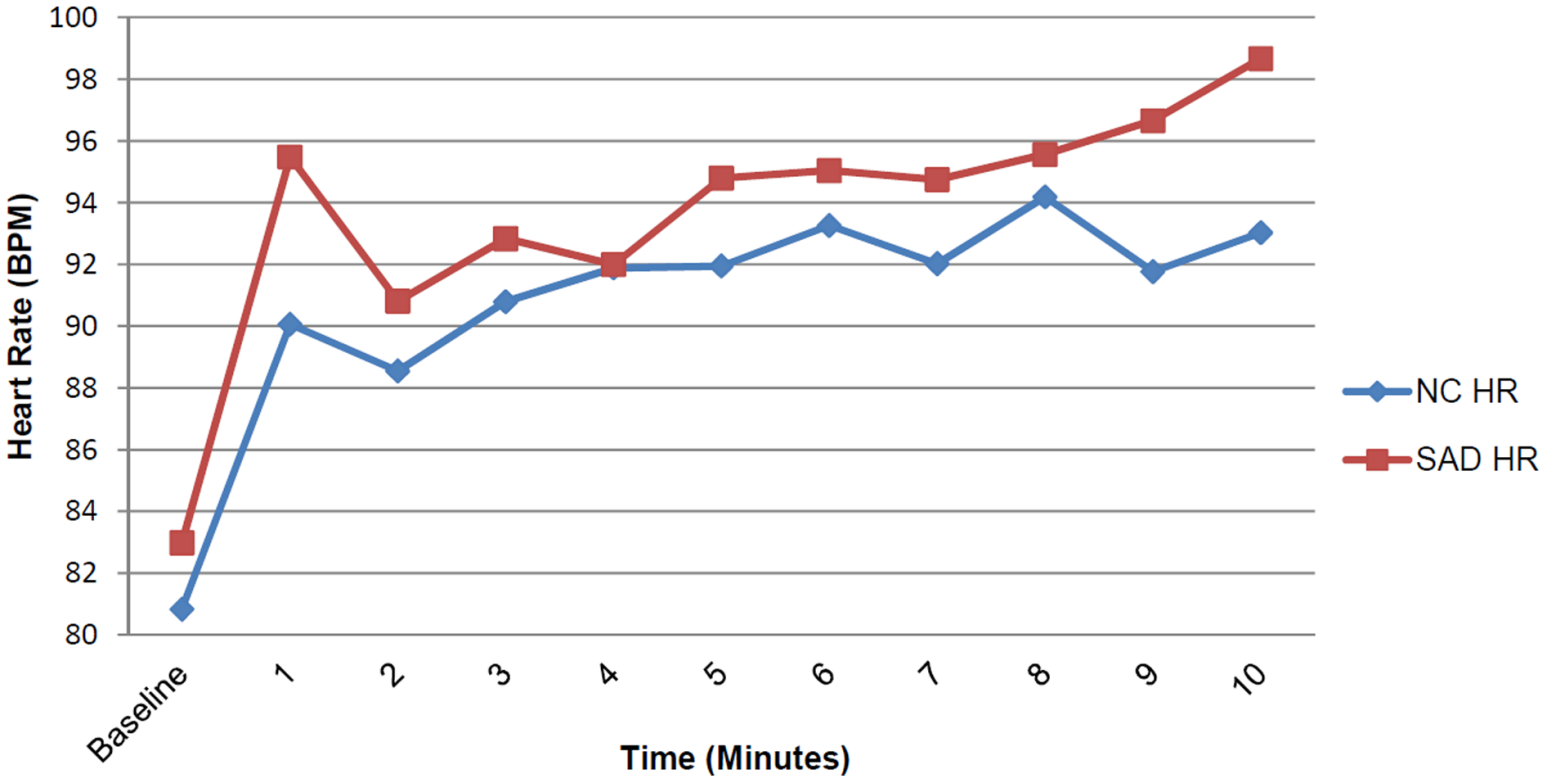
Mean heart rate during the Social Interaction Task. The plot depicts the mean heart rate for the groups from the baseline assessment through each 60-second interval of the Wii task.

During the SIT, SAD and NC participants did not differ in HR (Figure 1), *F*(1, 25) = .060, *p* = .808, η_p_^2^ = .002.

**Figure 2:**
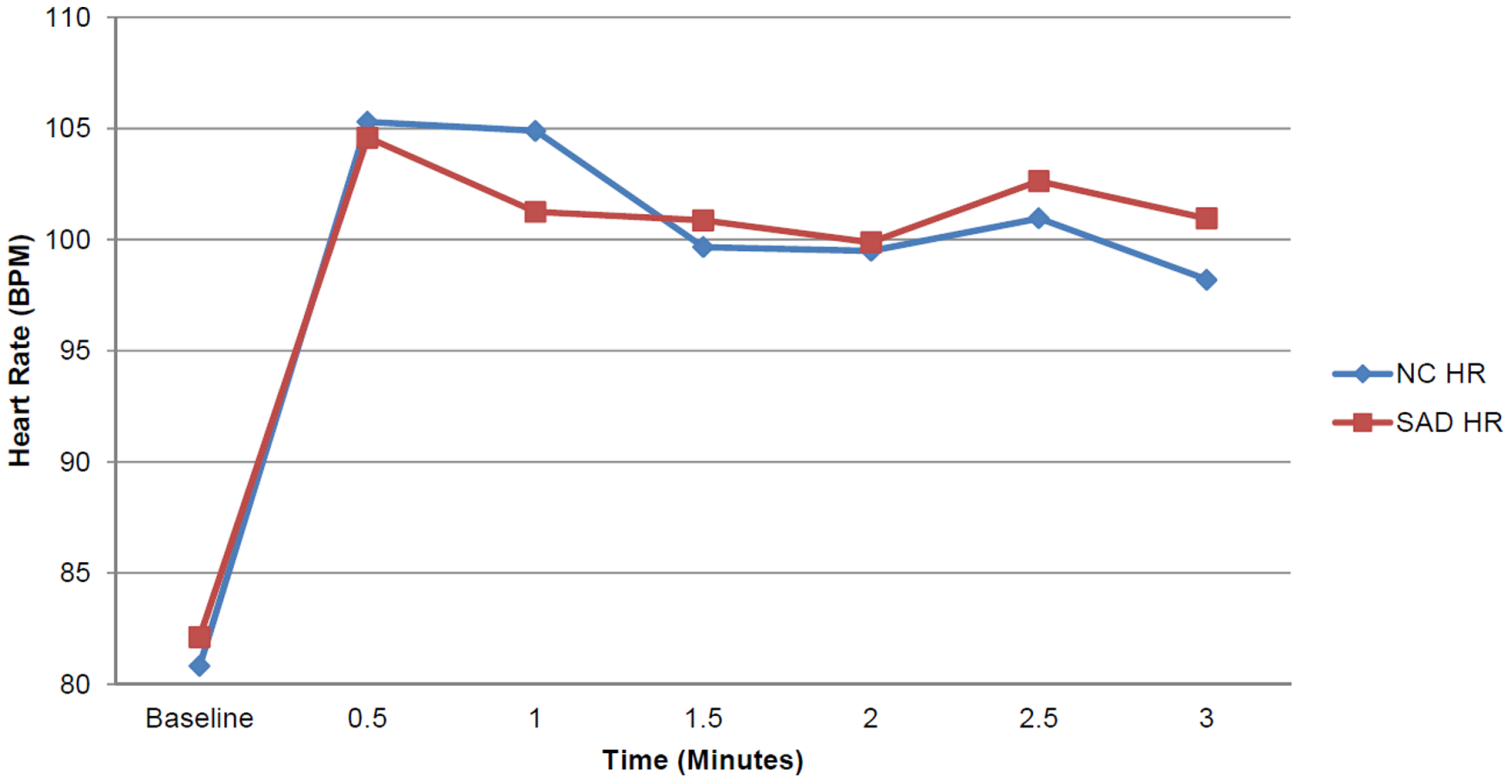
Mean heart rate during the Speech Performance Task. The plot depicts the mean heart rate for the groups from the baseline assessment through each 30-second interval of the speech task.

**Figure 3:**
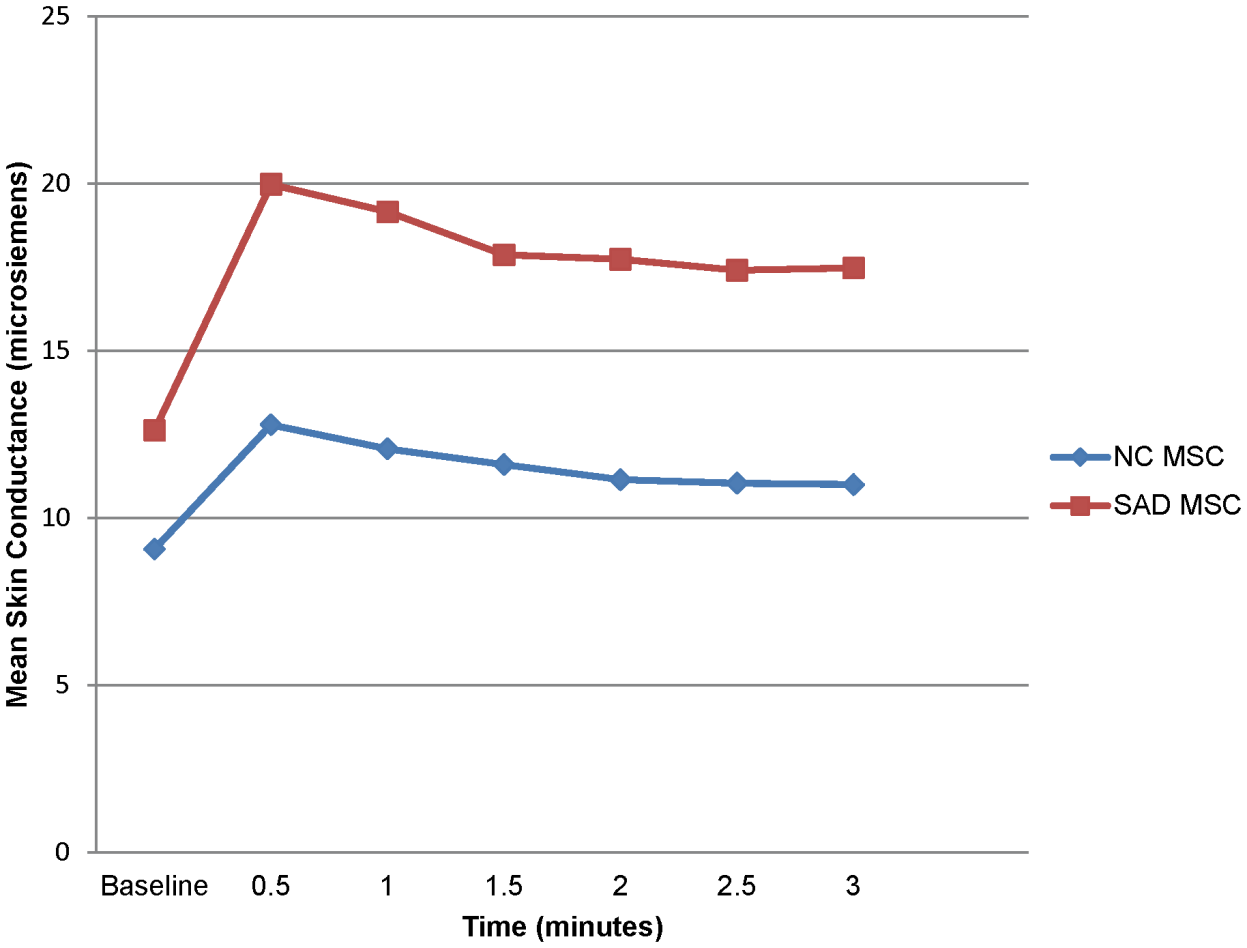
Mean skin conductance level during the Speech Performance Task. The plot depicts the mean skin conductance level for the groups from the baseline assessment through each 30-second interval of the speech task.

SAD and NC participants did not differ in HR during the SPT, *F*(1, 21) = .065, *p* = .802, η_p_^2^ = .003 (Figure 2); however, there was a significant group difference in MSC level, *F*(1, 22) = 6.524, *p* = .018, η_p_^2^ = .229. Follow-up analyses indicated that the participants with SAD had a significantly higher MSC level during every 30-second segment of the task, suggestive of heightened physiological arousal. A graphical representation of MSC level during the SPT is displayed in Figure 3. In addition, differences in SCR approached significance, *t* = −1.578, *p* = .058, as participants with SAD manifested more SCR than NC participants during the SPT.

## Discussion

The purpose of this investigation was to closely examine how SAD, during the time of adolescence, might impact daily functioning. The areas targeted for examination, which were based on gaps in the extant literature, included quality of sleep, social behavior in daily social situations, behavioral indicators of social skill during social and performance tasks, and physiological reactivity during these same tasks. The results indicated that in comparison to controls, adolescents with SAD do not experience ongoing sleep difficulties; however, this group does exhibit impairments in social skill and greater sympathetic activation during a public speaking task.

A multi-modal approach was used in this investigation to assess quality of sleep. Specifically, participants completed a subjective (e.g., self-report) and an objective (e.g., actigraph) measure of quality of sleep. Over the course of approximately one week, adolescents with SAD attained 7.36 hours of sleep on average per night and 94.29% sleep efficiency and did not report sleep difficulties, consistent with their NC peers and consistent with reports of normal sleep in adults with SAD [27]. Alfano and colleagues [33] found sleep difficulties in 27% of youth with SAD; however, that sample was considerably younger (mean age of 9.6 years). Quality of sleep in youth with SAD warrants further investigation to determine whether it may differentiate youth with SAD from youth with other anxiety disorders—namely generalized anxiety disorder and separation anxiety disorder—where poor sleep is self-reported and/or objectively observed to occur with much greater frequency [31], [33], [40], [41].

Adolescents with SAD do not report high levels of general arousal on a daily basis. Rather, their anxiety is elicited/precipitated by situations where there is a high likelihood of negative evaluation. Thus, it appears logical that, for adolescents with SAD, sleep disruption might not occur in the absence of an upcoming distressing event. In an attempt to address this issue, the actigraphy sleep data were also examined qualitatively while referencing anxiety-provoking events in the daily anxiety journal, as suggested by Chorney and colleagues [32]. Unfortunately, the timing of this investigation did not allow for assessment of all participants during times of stress (e.g., national testing exams such as the SAT). We did, however, attempt to examine sleep data in those days listed in the diary as containing a stressful event. There was no obvious relationship between either the frequency or the type of anxiety-provoking social activities reported and quality of sleep among participants with SAD.

Consistent with prior investigations [19], [25], adolescents with SAD reported significantly more distress (i.e., anxiety) during a social interaction and a public performance task. In contrast to the prior investigations, this study examined molecular social behaviors that contribute to the overall ratings of reduced social effectiveness described in the prior literature. Although the omnibus test for the SIT coded behaviors was not significant, analyses of the individual behaviors revealed that adolescents with SAD appeared less engaged socially during the SIT, as they asked significantly fewer questions and required significantly more confederate prompts to engage in conversation. Given that these prompts were delivered only after 2 minutes of silence from participants, these findings are consistent with prior observations of infrequent spontaneous comments among adolescents with SAD [42].

During the speech task, adolescents with SAD delivered speeches that were much shorter in duration, despite discussing nearly the same number of topics as NC adolescents. Thus, adolescents with SAD progressed quickly through several different speech topics, almost as if they were *naming* the topics rather than *discussing* them in detail. Additionally, and consistent with prior investigations in children [43] and adults [44], adolescents also exhibited significantly greater speech latency and were significantly more likely (50% to 0%) to escape the speech task. Collectively, the behavioral findings may be indicative of social inhibition or a suppression of social skill by anxiety, consistent with findings in the adult SAD literature [45]. Alternatively, these findings may provide further support for core deficits in social skill. Some empirical data suggests that at least some youth with SAD have underlying social skill deficits that remain even when anxiety is decreased [17], [46]. In a recent study, children with SAD demonstrated significantly less social knowledge on written social vignettes relative to children with generalized anxiety disorder and normal controls [47]. Accordingly, interventions for adolescents with SAD that combine social skill enhancement with anxiety reduction may be more effective [19], [48].

With respect to physiological assessment, this study expanded upon prior investigations by assessing two channels of physiology. HR, which is influenced by both sympathetic and parasympathetic activity, did not differ between groups during either task, consistent with prior physiological assessments of socially anxious adolescents [21], [22] and adults with SAD [49], [50]. With respect to cardiovascular reactivity, adults with SAD were found to exhibit increased systolic blood pressure reactivity during a speech task [44], but blood pressure was not assessed in this investigation.

Throughout the speech task, adolescents with SAD manifested significant autonomic arousal as measured by higher mean skin conductance levels. EDA is a measure of sympathetic (i.e., fight-or-flight) activation; therefore, this observation is consistent with other reports of elevated subjective distress and self-perceived increased physiological reactivity during speech tasks among adolescents with SAD [20], [21], [22]. The divergent physiological outcomes may be due to the different autonomic systems that influence these channels of physiology. While both HR and EDA are stimulated by the sympathetic nervous system, HR is also affected by the parasympathetic nervous system, which generally works to reverse the sympathetic response.

Finally, with respect to daily social activities, adolescents with SAD reported fewer activities completed, more frequent anxiety-provoking situations and greater avoidance of these situations. The social situations that were distressing to participants with SAD included speaking to strangers, participating in class, asking the teacher for help, assertiveness opportunities (e.g., asking someone to change their behavior) and interacting with a group. Similar to children with clinically-significant social anxiety [51], journal reports indicate that adolescents with SAD are faced with socially distressful events frequently. On average, participants with SAD completed reported 15 distressing and 6 avoided daily social events. Collectively, these findings suggest that the distress experienced by adolescents with SAD is likely to discourage further social activity, leading to avoidance that hinders socialization during a stage of development when socializing and establishing a social network in various settings are critical [52].

The present investigation is not without limitations. First, there was an unequal ethnic and male-to-female distribution between groups. Although it is unclear how ethnic differences may have influenced the results, there are physiological differences between sexes [53], [54] that may have influenced the findings. Additionally, the participants' stage of pubertal development was not assessed. Puberty is a time during which rapid hormonal changes associated with physiological and sleep pattern alterations occur that vary by sex [52], [54], [55], [56], [57], and age is not always the best indicator of progression through puberty. Third, the number of subjects was lower than desired, which was particularly evident in the SIT analyses; however, sufficient power was available to detect significant differences in a range of effect sizes (ηp^2^ = .128 to .526). Nevertheless, larger samples would be important in replication efforts. Finally, the SIT was designed to simulate a more typical social situation encountered by adolescents than the turn-based role-play tasks used in prior assessments of social skill; however, the task may have not demanded much social interaction because the participants' attention was drawn to the video game. Even so, participants with SAD reported significantly greater distress during the SIT and behavioral differences were observed, indicating that the task was effective at simulating an actual social situation. Additionally, the format of the SIT did not allow for assessment of EDA, which appears to be an important indicator of physiological distress. Playing the Wii requires hand movements, which in turn produces so many movement artifacts as to render the data uninterpretable. Future investigations may improve upon the SIT used here by designing an analogue social situation that minimizes movement artifact (e.g., social interaction while playing a board game).

Despite these limitations, the data from this investigation provide important contributions to the current understanding of SAD in adolescents. This study was the first attempt to identify specific behavioral indicators of social functioning and examine quality of sleep in adolescents with SAD using diverse assessment strategies. Future studies may build upon the present design by controlling for sex and ethnic differences through study recruitment. Furthermore, assessment of sleep to identify circumstances under which sleep difficulties develop in socially anxious adolescents. It may also be prudent to assess sleep during specific times of the school year that are likely to create stress for this group (e.g., standardized testing or first week of a new school). Furthermore, the molecular social behaviors observed during the analogue social situations here begin to clarify the specific difficulties that may contribute to impaired social functioning in this group. The behaviors present a picture of reduced effectiveness during public performance and a limited ability to engage in social interaction with an unfamiliar person. Interventions for this group that target formal public speaking and unstructured social interaction skills, in addition to anxiety reduction, may provide a more powerful intervention in the treatment armamentarium for adolescent SAD.
